# Genetic literacy among primary care physicians in a resource-constrained setting

**DOI:** 10.1186/s12909-024-05110-0

**Published:** 2024-02-13

**Authors:** Pascale E. Karam, Lina Hamad, Mohamed Elsherif, Khalil Kreidieh, Ghunwa Nakouzi, Khalil El Asmar, Tamar Kabakian-Khasholian, Dany Assaf Curi, Soha N. Yazbek

**Affiliations:** 1 Department of Biochemistry and Molecular Genetics, Faculty of Medicine, American University of Beirut, Beirut, Lebanon; 2 Department of Pediatrics and Adolescent Medicine, American University of Beirut Medical Center, Beirut, Lebanon; 3 Department of Epidemiology and Population Health, Faculty of Health Sciences, American University of Beirut, Beirut, Lebanon; 4 Faculty of Medicine, American University of Beirut, Beirut, Lebanon; 5 Hudson Alpha Clinical Services Lab, LLC, Huntsville, AL USA; 6 Department of Health Promotion and Community Health, Faculty of Health Sciences, American University of Beirut, Beirut, Lebanon; 7 Department of Pediatrics, Division of Hematology and Oncology, Saint George Hospital University Medical Center, University of Balamand, Beirut, Lebanon

**Keywords:** Genetics, Health literacy, Primary care, Continuing education, Knowledge, Healthcare practice

## Abstract

**Background:**

Genetic literacy among primary healthcare providers is crucial for appropriate patient care with the advances in genetic and genomic medicine. Studies from high-income countries highlight the lack of knowledge in genetics and the need to develop curricula for continuing professional development of non-geneticists. Scarce data is available from resource-constrained countries in Middle East and North Africa. Lebanon is a small country in this region characterized by high rates of consanguinity and genetic disorders like several surrounding countries, such as Jordan, Syria, and Turkey.

**Methods:**

The primary aim of this study assessed the genetic literacy, self-perceived and actual knowledge as well as practices among primary care providers in Lebanon. The secondary aim identified their educational needs and proposed evidence-based continuing education programs. A cross-sectional survey-based study, using a self-administered questionnaire, was conducted targeting physicians from Family Medicine, Obstetrics and Gynecology, and Pediatrics. The questionnaire was divided into five sections: demographics, familiarity with genetic tests, self-reported and actual knowledge, genetic practices, and educational needs. Statistics were performed using SPSS v24. The Chi-square test was used for independent variables. Differences between mean scores were measured using paired sample t-tests for groups of two levels and one-way ANOVA for more than two. Multiple linear regression was used to study the variables associated with the knowledge score while controlling for other variables.

**Results:**

The survey included 123 physicians. They were mostly familiar with karyotype as first-tier genetic test. Although 38% perceived their knowledge as good, only 6% scored as such in knowledge assessment. A better knowledge score was observed in academic institutions as well as in urban settings (*p*<0.05). One third never ordered any genetic testing, mostly due to poor knowledge. Almost all (98%) were ready to attend continuing professional development sessions in genetics.

**Conclusion:**

Our findings show the need to improve genetic literacy among healthcare frontliners, focusing on remote regions and nonacademic centers in Lebanon, a model for other resource-constrained country in the Middle East and North Africa region. This study advances recommendations for evidence-based genetic continuing education programs and highlighted the role of that the few genetic specialists can play in their successful implementation.

**Supplementary Information:**

The online version contains supplementary material available at 10.1186/s12909-024-05110-0.

## Introduction

Genetics and genomics have rapidly expanded to become an integral part of healthcare [1], requiring basic genetic literacy from primary care providers (PCP) worldwide [2]. General practitioners, family medicine, pediatric and adolescent physicians, in addition to obstetricians and gynecologists are the frontliners PCP dealing with patients at risk for, or with an underlying genetic disorder. As such, PCP need at least a basic level of knowledge in genetics to deliver the appropriate quality of care. The recent advances in genetic and genomic technologies facilitated the diagnostic confirmation, prevention and management of these disorders in clinical practice. Furthermore, the progressive reduction in cost of genetic testing, including whole exome, whole genome and mitochondrial DNA sequencing [3], made these tests more readily available to healthcare professionals in their daily practice. Timely and appropriate choice of testing is crucial for patients affected with genetic disorders; the main challenge resides in the lack of genetic literacy among non-genetic clinicians impacting their accurate utilization of genetic testing and subsequently, patients’ diagnosis and management.

Knowledge, attitudes and abilities of PCP and medical students assessed in some European and Asian countries [4–6] as well as in the United States [2] and Canada [7], highlighted the lack of knowledge of primary care providers in the field of genetics. Furthermore, the need to develop genetic curricula for continuing professional development of non-geneticists was also recognized even before the genomic medicine era [8]. Core competencies for PCP were described almost two decades ago in the United States [9, 10] and an online genetics and genomic course was created by The American College of Medical Genetics and Genomics to fill this gap for PCP [11]. In a recent review, Ong et al. [12] highlighted the need to assess genetic literacy and attitudes of PCP towards clinical genetics services before adopting any educational intervention and possible “shared care models” with other genetic healthcare providers.

Studies of genetic literacy among PCP from Middle East and North African (MENA) countries are scarce despite the high prevalence of genetic diseases [13], and consanguinity rates in this region [14] which predisposes for autosomal recessive disorders. In a review by Nakouzi et al. [14], the inheritance pattern of genetic disorders was mostly autosomal recessive in 67%, followed by autosomal and X-linked disorders in 17% and 6%, respectively. A list of 378 types of genetic diseases were reported among Lebanese patients, in addition to a large number of chromosomal abnormalities. The most common genetic diseases in Lebanon include congenital malformations and chromosomal abnormalities (trisomy 21, Turner syndrome.), followed by metabolic disorders (phenylketonuria, medium-chain acyl-CoA dehydrogenase deficiency, mitochondrial diseases.).

Few high-income MENA countries like Saudi Arabia and Qatar conducted assessments of medical students and PCP, showing the imperative need to integrate genetics in residency programs [15] and the establishment of graduate genomic studies [6]. Lebanon is a small country in the MENA region, with limited resources and a high prevalence of genetic diseases [13], like several other countries in the region Jordan [16], Syria [17], or Turkey [18]. Despite the availability of highly specialized healthcare providers in Lebanon, few geneticists are recognized [14]. There is no certification system for healthcare providers specializing in genetic medicine or counseling in Lebanon. Furthermore, less than ten healthcare professionals in the field of genetics are practicing mainly in five academic institutions in Lebanon [14].

In contrast, several genetic testing services are available throughout the country, mostly outsourcing genetic tests to reference laboratories in Europe or the United States. Subsequently, genetic testing is ordered and interpreted frequently by primary care providers who may not be sufficiently trained to diagnose and manage patients with genetic diseases in resource-constrained countries [19]. The most ordered genetic tests by primary healthcare providers in Lebanon included initially karyotype and single gene testing but, more recently, there is an increased interest in ordering gene panels and even genome-wide testing.

The primary aim of this study was to assess the genetic literacy, self-perceived and actual knowledge, as well as practices among PCP in Lebanon. The secondary aim was to identify the PCP educational needs and propose measures that can be adopted in resource-constrained countries with high prevalence of genetic disorders.

## Methods

This study was conducted between February 2019 and August 2019, before the economic crisis in Lebanon. The targeted population consisted of PCP from various disciplines Family Medicine (FM), Obstetrics and Gynecology (OBGYN), and Pediatrics (PED). An online survey was sent to all registered healthcare in these specialties in the country, as well as reminders by phone messages. Flyers were also distributed at seminars, meetings, and conferences planned by national medical societies, with the option to fill out a printed version of the questionnaire. This study was approved by the Institutional Review Board at the American University of Beirut, Lebanon. Informed consent was obtained from all participants in this study.

The questionnaire was divided into five sections to report on demographics, familiarity with genetic tests, self-reported and actual genetic knowledge, assessment of practice and educational needs. The developed questionnaire was adapted from Clyman et al. [20], and the GPGeneQ by Flouris et al. [21], both of which were validated. In the demographics section, the regions were characterized by the authors as urban and rural, based on the specific district name responses. Urban areas included Beirut, Mount Lebanon, and North Districts, while South and Bekaa were considered as rural.

Familiarity with genetic tests was self-rated. The actual knowledge assessment included 30 questions. The average score of all questions was calculated to range from − 1 (all questions were wrongly answered) to 1 (all questions were correctly answered). A score below zero was considered as poor knowledge, between 0 and 0.5 average knowledge and above 0.5 good or adequate. Genetic practice evaluation included taking full family history, ordering genetic tests, discussing results with patients and referrals to genetic clinics. Educational needs were self-reported and prioritized. The questionnaire was pilot tested with a small number of physicians in academic institutions for relevance, perception, clarity, and ability to achieve participation. Their feedback was considered for the final version.

### Sample size

We considered the total number of primary care physicians (PCPs) registered with the Lebanese order of physicians, which stood at 2753. We utilized a confidence level of 95% with a standard Z-score of 1.96 and estimated a conservative response rate based on previous literature and preliminary consultations. The sample of 1200 physicians contacted represents approximately 43.6% of the total PCP population. This proportion was chosen to exceed the minimum sample size that would account for anticipated variability within the population. With a response rate of 10.3%, the questionnaire was filled by 124 physicians, which was sufficient to maintain the power of the study. Only one participant was excluded due to incomplete gender, age and subspecialty data. The margin of error for the response rate was calculated to be approximately 5.23%, indicating that the results are sufficiently precise to reflect the views of the larger PCP population within a reasonable confidence interval. The sample size was further justified post hoc by the finite population correction, which indicated that the margin of error remained within an acceptable range, confirming that the sample of 123 physicians is statistically sufficient for the objectives of this cross-sectional survey.

### Statistical analysis

Analysis was performed using SPSS v.24. Descriptive statistics such as frequencies and proportions were reported for categorical variables while mean and standard deviation were reported for numerical variables. Comparing and contrasting demographic and practice characteristics were done using the Chi-square test for independent variables. Differences between mean scores were measured using paired sample t-tests for groups of two levels and one-way ANOVA for more than two.

For the knowledge section, a score was calculated to reflect the actual knowledge where every correct answer was given a positive mark, while a mark was deducted for every wrong answer. Results were expressed as a percentage of those who responded to each item (valid %). The relationship between knowledge score and participants’ age was tested using Pearson’s correlation. Multiple linear regression was used to study the variables associated with the knowledge score while controlling for other variables. Questions related to the genetic practices were summarized and presented in the form of numbers and percentages. A *p*-value of less than 0.05 was considered statistically significant.

It is important to mention that although the total number of participants was 123, not all the questions in the questionnaire were answered by all the participants. The value ranged between 110 and 124 answers/question. This is why we used percentages to normalize the data and make comparisons on a relative scale.

## Results

### Demographics

**Fig. 1:**
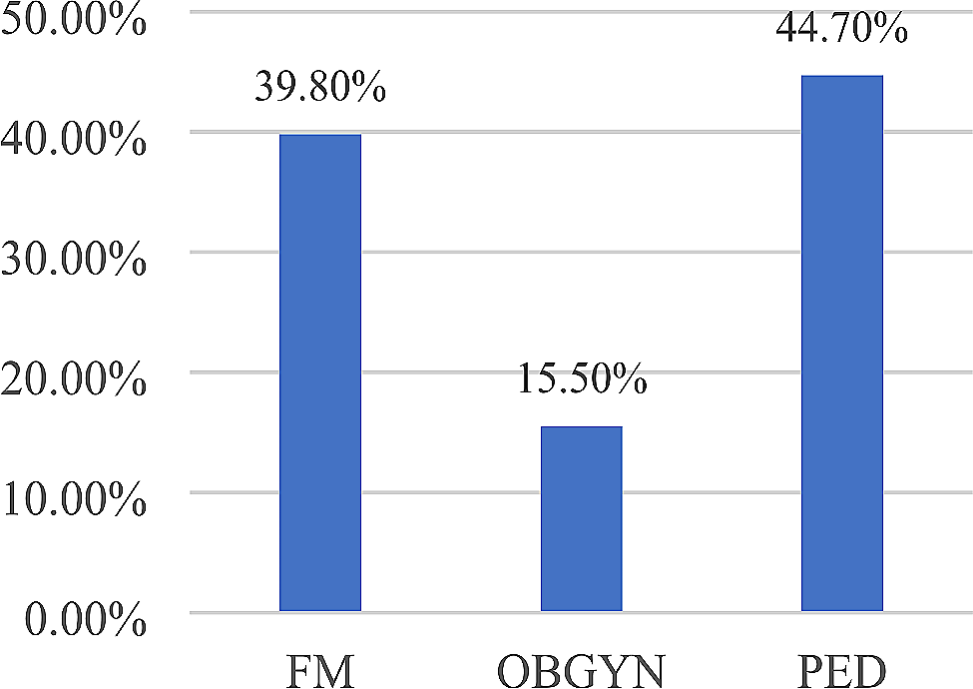
Distribution of physicians by specialty. (Abbreviations: FM: Family Medicine, OBGYN: Obstetrics and Gynecology, PED: Pediatrics and Adolescent Medicine)

**Fig. 2:**
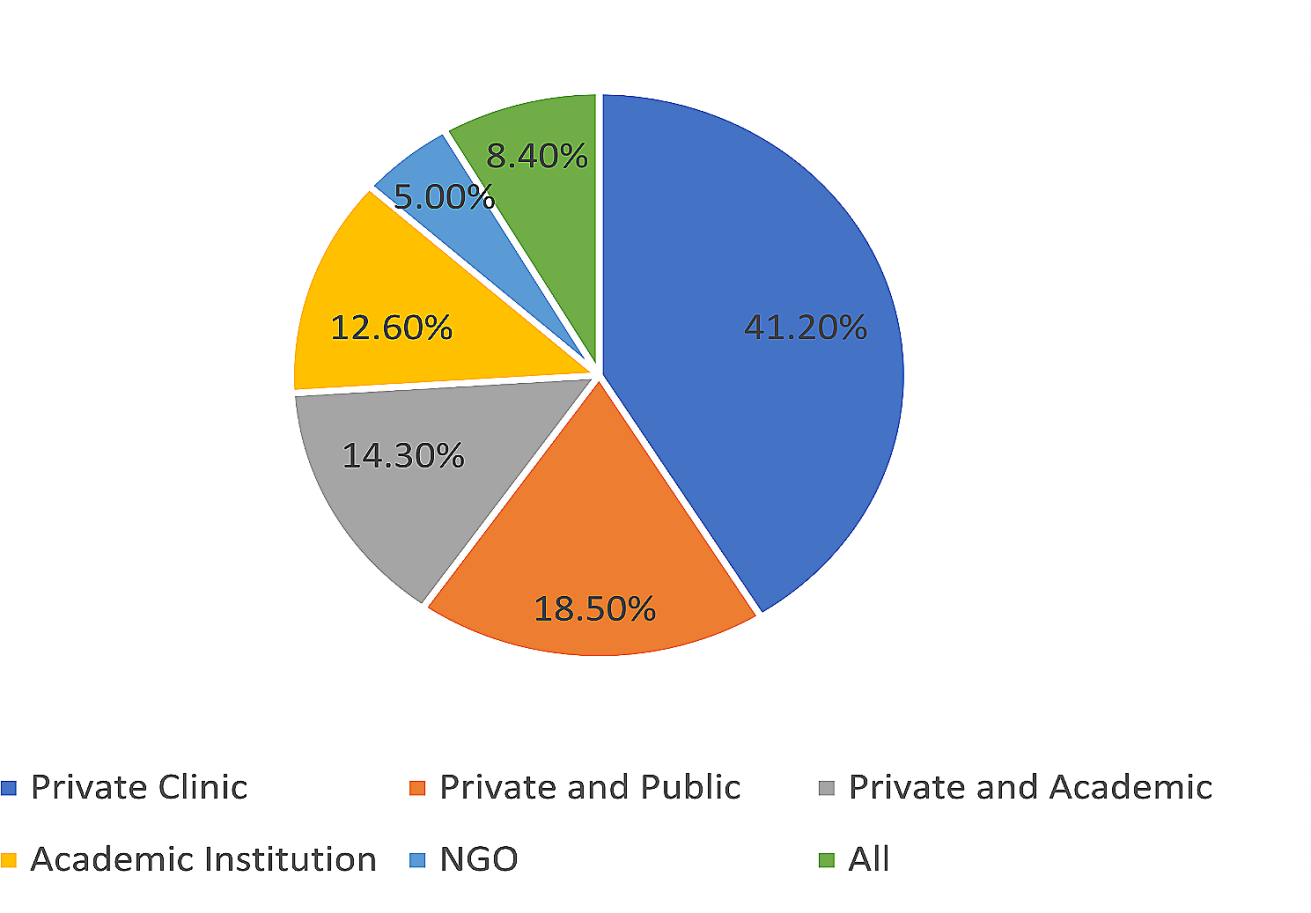
Distribution of surveyed physicians among healthcare sectors

A total of 1200 physicians were contacted out of 2753 registered at the Lebanese order of physicians in the surveyed disciplines in the country. A response rate of 10.3% was obtained, with 123 physicians included in the study. Most participants were males (64%). The mean age was 47.8 ± 10.8 years and the mean years of practice was 17.7 ± 10.9 years. Pediatricians were mostly represented (Fig. 1). Participants were recruited from districts across all Lebanon. Distribution among healthcare sectors was variable, mainly from the private non-academic sector (Fig. 2) (Table 1).

**Table 1:** Demographic characteristics of the respondents

Variables		N	%
***Specialty***	**FM**	49	39.8
	**OBGYN**	19	15.5
	**PED**	55	55
	**Total**	**123**	100
***Gender***	**Males**	79	64
	**Females**	44	36
	**Total**	**123**	100
***Healthcare sector***	**Private clinic**	49	41.2
	**Private and public**	22	18.5
	**Private and academic**	17	14.3
	**Academic**	15	12.6
	**NGO**	6	5
	**All sectors**	10	8.4
	**Total**	**119**	100
**Mean age**	47.8 ± 10.8		
**Mean years of practice**	17.7 ± 10.9		

### Familiarity with different genetic tests and techniques

**Fig. 3:**
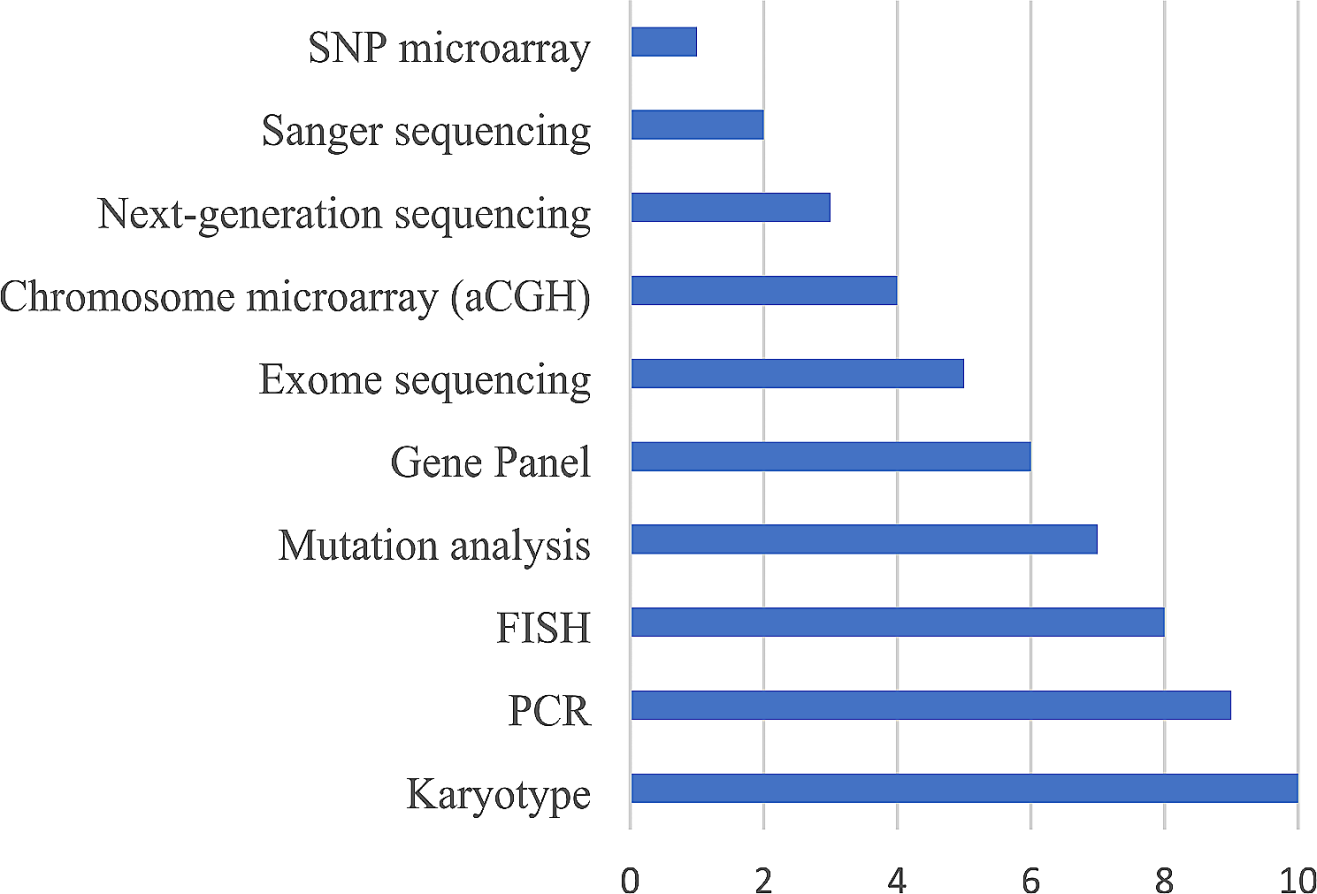
Familiarity of primary care providers with genetic tests and techniques. (Scoring from 1 to 10: from lowest to highest familiarity)

Familiarity with different genetic tests was ranked from 1 to 10, the highest score reflecting the highest familiarity with the testing modality. Physicians were mostly familiar with karyotype testing and knew much less about single nucleotide polymorphism microarray (Fig. 3).

### Genetic knowledge

**Fig. 4:**
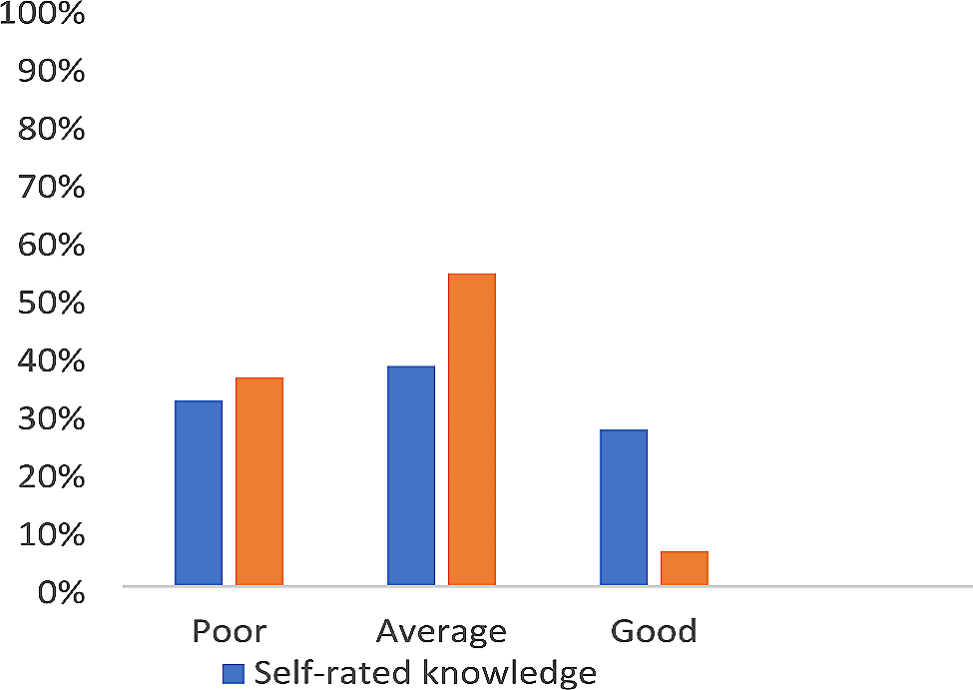
Self-rated and assessed genetic knowledge of 123 primary care providers

**Fig. 5:**
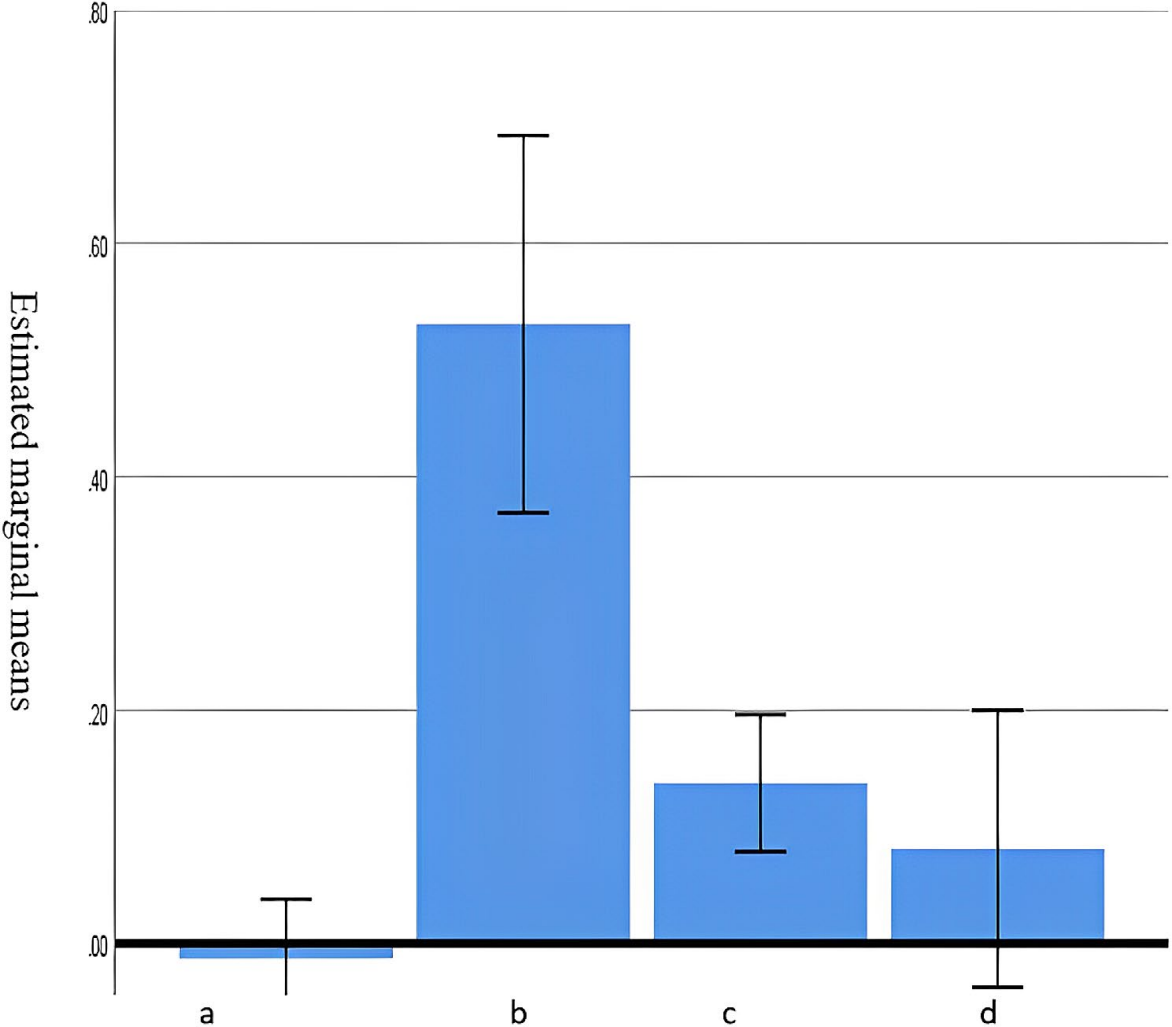
Average score of genetic knowledge scopes among 123 primary care providers (a: Basic genetic knowledge, b: able to recognize a genetic syndrome, c: knowledge of appropriate genetic testing, d: knowledge of appropriate plan of care)

Participants were asked to rank their own knowledge of genetics. Although 38% (out of 119 respondents) perceived their knowledge as good, only 6.0% scored more than 0.5 and had most questions answered correctly (Fig. 4). The minimum achieved score was − 0.46, while the maximum score was 0.68 (median 0.18, mean 0.16). Actual genetic knowledge assessment revealed that 32% (out of 123 respondents) achieved a poor knowledge score of zero or less and 54% had an average knowledge score. The highest score was observed for recognizing a syndrome or disease while the lowest score was for general basic information (Fig. 5).

There was a statistically significantly better knowledge score between PCP practicing in academic institutions versus those in non-academic institutions (*p*-value = 0.001). Surprisingly, there was no difference in the knowledge score among physicians reporting receiving continuing medical education credits in genetics compared to those who did not.

The correlation between the average knowledge score, age, and years in practice showed that higher age and years in practice were associated to lower knowledge score (*p*-value < 0 0.001). Furthermore, scores across practice districts showed that PCP practicing in urban regions had significantly higher knowledge in comparison to those in rural areas (*p*-value < 0 0.001). Multiple linear regression analysis revealed that the relationship between knowledge score and age, but not district of practice, was dependent on other variables that affected the overall association (Table 2).

**Table 2:** Multiple linear regression analysis for different factors associated with knowledge score. Abbreviations: St. error: standard error, CI: confidence interval, CME: continuous medical education, FM: family medicine, OBGYN: obstetrics and gynecology, PED: pediatrics

Variables	Coefficient	St. error	*p*-value	95% CI	
Age	-0.004	0.002	0.165	-0.008	0.001
Gender (Female)	0.027	0.055	0.623	-0.083	0.137
Receiving CME (Yes)	0.066	0.062	0.294	-0.058	0.189
Having practice in academic institution (yes)	0.101	0.063	0.115	-0.025	0.226
Having a clinical geneticist in their practice (Yes)	0.058	0.075	0.442	-0.092	0.208
*Specialty*					
FM	reference				
OBGYN	0.046	0.080	0.570	-0.113	0.204
PED	0.040	0.061	0.517	-0.082	0.161
*District*					
Urban	reference				
Rural	-0.256	0.087	0.004	-0.430	-0.082

### Genetic practices

A full family history for new patients, including second degree relatives such as grandparents, uncles, and aunts was shown to be collected only by 8% of PCP (out of 121 respondents). One third of PCP never ordered any genetic testing attributing this to multiple reasons, including: poor knowledge in the field (35%), considering genetic tests out of their scope of practice (22%) and lack of genetic clinics for referral (17%). Discussion of genetics test results confidently with patients was provided by PCP who self-rated their knowledge as good, in contrast to those who self-rated their knowledge as poor (*p*-value < 0.001). The majority of PCP who reported referring to genetic clinics had a good actual knowledge score (above 0.5).

### Perceived educational needs

Participation in future genetic training was explored, and almost all physicians (98%) showed readiness to attend either workshops, online modules, monthly seminars, or all of these. Their scored priorities from highest to lowest were training in recording family history (*n* = 66), recognizing genetic conditions (*n* = 64), understanding gene therapy (*n* = 62), use of computer database for clinical diagnosis (*n* = 61), new laboratory techniques (*n* = 54), and interpretation of DNA test results (*n* = 49). The least priorities were for trainings in genetic counseling (*n* = 47) and mitochondrial inheritance (*n* = 28).

## Discussion

This study sheds the light on the gap between genetic literacy among PCP and the increasing needs for continuing professional development, in the era of genomic medicine, particularly in a resource- constrained country with high prevalence of hereditary disorders. Surveyed physicians included healthcare providers likely to encounter patients with genetic disorders in their daily practice. The response rate of 10.3% falls within reported rates by other surveys [22, 23]. Physicians were equally distributed and represented across districts which decreases the non-response bias. The majority of PCP were acquainted with basic genetic tests like karyotype, which may be related to their poor knowledge about the new genetic techniques as reported by Haga et al. [24]. The most alarming result of this survey is the unjustified self-confidence of PCP who reported their knowledge as adequate compared to their actual knowledge. Similarly, limited actual knowledge of genetics was shown among healthcare providers in general, whether in high-income countries like the United States and Canada [25, 26] or in resource-constrained countries like Brazil [27]. This inflated self-ranking of knowledge may be attributed to receiving continuing medical education in genetics which improved the perception of being knowledgeable rather than the actual computed knowledge. This draws attention to the importance of post-training assessment and evaluation. Furthermore, this false perception of being knowledgeable will have a negative impact on the quality of genetic practice and the recommendations offered to patients and their families.

The genetic knowledge disparity between freshly graduated and senior physicians reflects the importance of targeting training programs to physicians who received their medical training prior to the formal integration of genomics into the curriculum. Nevertheless, a recent study by Falah et al. [28] highlighted the need to integrate genetic education in primary care residency programs as well as in continuing medical education.

The geographical factor impacted genetic knowledge as well, with lower literacy scores among PCP practicing in rural and remote areas. As such, demographic and geographic differences in genetic literacy may play a major role in the disparities in patient care and may lead to major inequity among patients. Access to genetic experts is limited in rural communities, even in high-income countries like the United States [29]. In Lebanon, access to experts in the capital or central areas is limited due to transportation barriers and lower socioeconomic status in remote regions. Subsequently, patients in rural areas with a high burden of genetic disorders and consanguinity rates [14, 30] depend solely on the knowledge of their local physicians showing less genetic literacy. Limited genetic practices and knowledge in addition to lack of access to referral services represent major barriers also delineated by previous studies conducted among non-geneticist healthcare providers in several countries [2]. The educational priorities of the physicians reflected their need for practical topics, from collecting family history to recognition of genetic disorders and indications for referral to genetic services, similarly to PCP in other countries [2].

### Recommendations

The establishment of continuing professional development genetic modules targeting primarily senior PCP and those practicing in rural areas will improve patient care with access to personalized genomic medicine. Most genetics educational programs for non-genetic healthcare professionals were shown to be effective [31]. The insight of clinical genetic professionals into teaching components would be beneficial as well [32]. A multi-step approach could also be adopted [26]. Moreover, a mixed modality educational program can be proposed, starting with an in- person seminar to elicit the interest and awareness of the physicians followed by weekly online sessions. At the end of the program, a closing workshop would be offered. The false perception of sufficient knowledge among PCP can be addressed through self-assessment quizzes within the training program. The educational material needs to cover at least basic needed genetic skills such as taking family history, suspecting genetic conditions, new laboratory techniques, understanding DNA results, and available treatments.

Furthermore, telemedicine is increasingly being used with high level of patient satisfaction to address the challenges of poor access to genetic services in rural areas and PCP lack of knowledge [30]. This solution may be adopted as a transitional or back-up solution, pending the achievement of satisfactory genetic literacy among PCP.

## Conclusion

In conclusion, despite their limited number, genetic healthcare professionals in Lebanon must play a pivotal role in the successful implementation of the above recommendations. They can do so by providing the mentioned educational programs and training sessions to PCP, helping them understand the basics of genetics and its applications in medicine. Furthermore, in the context of telehealth, genetic specialists can serve as consultants offering guidance to PCP on when and how to incorporate genetic testing and counseling into their practice. They can help as well interpret complex genetic test results and provide recommendations for further evaluation or management based on the findings. They can also share research findings with PCP through academic seminars, helping them stay informed about the latest advancements in the field.

This is the first study from Lebanon and other resource-constrained countries in MENA region exploring genetic literacy among PCP and proposing solutions to overcome the knowledge gap and poor access to clinical genetic services. This study provides evidence of major genetic knowledge shortage among PCP in Lebanon and the need of organized genetic educational programs. Future studies assessing the impact and effectiveness of these educational programs are needed.

### Electronic supplementary material

Below is the link to the electronic supplementary material.

Supplementary Material 1

## Data Availability

The datasets generated during and/or analyzed during the current study are available from the corresponding author on reasonable request.
