# Supplementary material for: Genetic literacy among primary care physicians in a resource-constrained setting
Source: BMC Med Educ. 2024 Feb 13;24:140. doi: 10.1186/s12909-024-05110-0 (PMC10863164; doi:10.1186/s12909-024-05110-0)
Supplement: Supplementary file 1 — Supplementary Material 1 [file 12909_2024_5110_MOESM1_ESM.docx]

Supplementary Document 1

**PCP Questionnaire**

This questionnaire included five sections:

1. Demographics
2. Familiarity with genetic tests
3. Self-reported Knowledge
4. Actual Knowledge
5. Perceived Education Needs

**Section I:** **Demographics**

In this section, data collection included: age, gender, years in practice, and specialty as well as location of practice (district) and number of clinics attended. Specifying whether in private or public health clinics (NGO or Ministry of Health), having practice in academic institution, and having a clinical geneticist in their practice. Physicians were also asked if they received any continuing medical education about genetics and if they refer to geneticists.

**Section II: Familiarity with different genetic tests**

How familiar are you with the following genetic techniques used?

|  |  |  |  |  |
| --- | --- | --- | --- | --- |
| Karyotype |  |  |  |  |
| FISH |  |  |  |  |
| PCR |  |  |  |  |
| Mutation analysis |  |  |  |  |
| Gene Panel |  |  |  |  |
| Sanger sequencing |  |  |  |  |
| Next-generation sequencing |  |  |  |  |
| SNP microarray |  |  |  |  |
| Exome sequencing |  |  |  |  |
| Chromosome microarray aCGH |  |  |  |  |

**Section III: Self-reported genetic knowledge**

**Questions**

- How would you rank your knowledge of genetics?
- How often do you complete a full family history (ie including 2nd degree relatives such as grandparents, uncles and aunts) for a new patient?
- Have you ever ordered any genetic tests? Which ones?
- How confident did you feel discussing the result with the patient?
- If you have never ordered tests, what was the main reason?

**Section IV- Actual knowledge**

**Questions**

- Normal chromosome complement

- Nucleotide components of DNA

- Gene Function

- Karyotype

- Basic genetic definitions (variant, polymorphism, mutation ..)

- Inheritance patterns

- Fetal risk calculations

- Genetic screening during pregnancy: chorionic sampling, serum markers

- Turner syndrome

- Cystic fibrosis

-Alzheimer`s disease

- Down syndrome

-Sickle cell anemia

- Genetic counseling

- Sudden infant death syndrome and medium chain acylCoA dehydrogenase deficiency (MCAD)

-Genetic predisposition for cancer

- Thalassemia carrier state

- Fragile X syndrome

- Newborn Screening for hypothyroidism, phenylketonuria, congenital adrenal hyperplasia, and cystic fibrosis

- Hemophilia

- Genetic clinical diagnostic tests

- Familial developmental delay and course of action

**Section V- Perceived Education Needs**

**Questions**

-If there were any training modules offered in genetics, would you attend?

- How would you prefer the training to be delivered?

- Specify your perceived educational priority in genetics: from lowest to highest for each of the following topics:

Training in recording family history, recognizing genetic conditions, mitochondrial inheritance, understanding gene therapy, use of computer databases for clinical diagnosis, new laboratory techniques, interpretation of DNA test results, trainings in genetic counseling.
